# Biomarkers come of age: PD1 in the frontline and cell cycle therapy swells the ranks of personalised therapy in the European Society of Medical Oncology (ESMO) congress, Copenhagen, 7–10 October 2016

**DOI:** 10.3332/ecancer.2016.703

**Published:** 2016-12-16

**Authors:** Will Davies

**Affiliations:** Cancer Intelligence, 154 Cheltenham Road, Bristol, UK BS6 5RL

**Keywords:** ESMO, checkpoint immunotherapy, lung cancer, bladder cancer, ovarian cancer, breast cancer, CDK, fulvestrant, PD-1, PD-L1, PARP inhibition, urothelial cancer

## Abstract

After years of trials, Programmed Death Ligand and Receptor targeting finally debuts as a firstline therapy in combination and as a single agent regimen at the 2016 European Society of Medical Oncology (ESMO) Congress. The meeting brought together 20,522 attendees, from over 120 countries, to share updates and novel technologies from a wide swathe of oncology research. This year’s theme, *From Disease Treatment to Patient Care*, was matched by abstract presentations starting from inception of care regimens to new standards of care in high-risk patient subgroups, to wellbeing of care providers, and finally the funding obstacles at each continental level.

## Essential checkpoints for PDL-1 passed

The biggest news of the conference came in as a burst of three publications on Sunday morning with doctors Martin Reck (Lung Clinic Grosshansdorf, Germany), Corey Langer (University of Pennsylvania, USA), and Fabrice Barlesi (Hôpital Nord, Marseille, France) presenting results from trials of pembrolizumab and atezolizumab as a firstline therapy against lung cancer.

Dr Martin Reck spoke first describing results from the KEYNOTE-024 trial. He stated that advanced non-small cell lung cancer patients with high PDL1 expression (tumour proportion score >50%), pembrolizumab improves overall survival (OS) with one year survival rates of 70% against 54% and improved median progression free survival (PFS) from six months to 10.2 months compared to platinum chemotherapy [1]

These results were matched by Dr Corey Langer [2], reporting on KEYNOTE-021, which found patients in the same setting who had not been assessed for PD-L1 expression and who went on to receive pembrolizumab alongside standard platinum chemotherapy showed an improved PFS rate (median 13.0 months versus 8.9 months) and a significantly greater objective response rate (55% versus 29%)

Dr Fabrice Barlesi gave similar results from the OAK trial of atezolizumab versus docetaxel in 1225 pretreated NSCLC patients. The finding was that of a 27% improvement in OS in the patients receiving azetolizumab regardless of their PD-L1 expression. For those in the top tertile of PD-L1 expression, the OS was 59% greater.

These results came within a half hour of each other along with the subsequent approval of atezolizumab by the US Food and Drugs Administration, so one might see this press conference will go down as a landmark moment for immunotherapy.

Further results presented over the next two days continued to hammer home the arrival of immunotherapies across indications. To this end Dr Arjun Balar (NYU Langone Medical Center, USA) and Dr Matthew Galsky (Mount Sinai, New York, USA) presented reports on the KEYNOTE-052 trial of firstline pembrolizumab and CHECKMATE-275 trial of nivolumab monotherapy for advanced urothelial cancer, and Dr Julie Graff (Knight Cancer Institute, USA) covered the trial of combining pembrolizumab and enzalutamide to treat the previously-considered-non-immunogenic prostate cancer. With such significant benefits seen in such a wide array of tumour sites and more results sure to come, the positive indications of PD-1 targeting almost seem to outstrip whether or not there is any PD-1/L1 detected.

One of the more innovative takes on the PD-1 pathway was discussed by Dr Omid Hamid (The Angeles Clinic, USA) who introduced phase I trials of MEDI0680 and durvalumab. By combining an anti-PD-1 and anti-PD-L1 antibody, Dr Hamid, achieved an 18% response rate in phase I trials, including one complete response (CR) and eight partial responses (PR) of which almost all have an ongoing response. With this pincer-manoeuvre, any sign of PD-1 or its ligand in tumours could be actionable as observed in upcoming trials of renal carcinoma.

Similarly, Dr Hardev Pandha (University of Surrey, UK) introduced results from the KEYNOTE 200 trial of pembrolizumab alongside oncolytic virotherapy. He described this as ‘lighting the fire’ of immune response and even introduced PDL-1 to the tumour thereby offering another means by which immune evasion could be outflanked.

CTLA4, not to be forgotten as an alternative checkpoint, gained further validation through the research presented by Dr Alexander Eggermont (Institut Gustave Roussy, France) from the EORTC 18071 trial [3]. His findings indicated that adjuvant ipilumumab significantly improved outcomes for high risk melanoma patients with OS at five years;11% higher in the ipilimumab arm than in the placebo arm (65% versus 54%). With ipilumumab having been approved for firstline therapy five years previously and now being verified in the adjuvant setting, its journey almost seems to be an inversion of that of PD-1. It may provide a course by which PD-1 charts its own development in the coming years.

## PARP inheritors

Waiting to take centre stage, with a similar swell of attention as PD-1 in recent years, poly-ADP ribose polymerase (PARP) inhibitors have already made an impression with the recent successes and adoptions of olaparib for breast and ovarian cancer. Expansion of PARP inhibitors now seems assured, i.e. following the presentation of data from the ENGOT-OV16/NOVA trial by Dr Mansoor Raza Mirza (Copenhagen University Hospital, Denmark) [4]. He assessed the response of patients with platinum sensitive recurrent ovarian cancer to either placebo or niraparib, heir-apparent to olaparib. Dr Mirza described the extent of patient benefits in terms of PFS as unprecedented in recurrent ovarian cancer with a difference of 21 months against 5.5 months for BRCA mutant patients. Even patients without BRCA mutations saw their PFS more than double, from 3.9 to 9.3 months.

Comparing this to the results from Dr Yung-Jue Bang (Seoul National University, South Korea) [5], who described the impact of olaparib alongside paclitaxel for gastric cancer patients, there was extension of PFS and OS but not to of statistical significance with a p value being 0.262.

## New targets and treatments for ovarian cancer

Dr Susana Banerjee’s (The Royal Marsden, UK) approach to recurrent ovarian cancer in the CORAL trial came from a similar hormone-targeting angle with abiraterone, a CIP17 upstream inhibitor of both androgen and oestrogen. Starting from a cohort of only 42 patients, of whom only one fully responded, Dr Banerjee notes a further 26% of patients showed clinical benefit of which 14% was prolonged. These proportions might improveshe said given better understanding of responder genotypes.

Another avenue in treating ovarian cancer came through research presented by Dr Judith Balmaña (Vall d’Hebron Instituto de Oncología, Spain) and Dr Cristina Cruz (Vall d’Hebron Instituto de Oncología, Spain) who approached trabectedin analogue lurbinectedin from opposite ends. Dr Balmaña reported on the OS of metastatic breast cancer patients with BRCA1/2 mutations at 20 months with BRCA2 mutations twice as likely to respond than BRCA1. She also noted patients who had not previously been treated with platinum had a 56% response rate compared to 26% for those who had been previously treated. For those who had previously been treated but had cisplatin resistant tumours, Dr Cruz described the utility of lurbinectedin in treating cisplatin resistant metastatic breast cancer, where there was no cross-resistance for the aforementioned PARP inhibition, with a 75% success rate.

With trials in PARP inhibition, lurbinectedin, and abiraterone ongoing, the overlap of one therapy to the resistance developing to another seems to be leaving fewer and fewer places for cancer cells to escape to, i.e. even for the previously untouchable indications.

## Success and setbacks for hormone therapy

On the subject of which, hormone signalling through the now ubiquitous hunt for familiar biomarkers—VEGF, EGF, etc—is proving indicative of action in more and more cancer types for more and more sites.

Dr Mark Kieran (Dana Farber Cancer Institute, USA) presented preliminary data of dabrafenib, a BRAF inhibitor, for the <10% of paediatric glioma patients with a BRAF V600 mutation. Paediatric brain tumours are typically associated with a high-wire act of tumour reduction through treatment without causing significant damage to developing brain tissue. With dabrafenib, Kieran describes successful treatment, with patients surviving and growing into healthy adults.

The objective response rate was 72%, with 23 out of 32 patients responding to the drug, of whom 13 patients had stable disease of at least six months’ duration, and 11 of them are still on the therapy. He also noted that either for a lack of environmental exposure or for another as-yet unknown mechanism, paediatric patients also did not exhibit the toxicity profile of adult melanoma patients who ran the risk of developing squamous cell carcinoma. Recruitment is ongoing for new trials in which dabrafenib will be paired alongside a MEK inhibitor and where aberrant signalling is strongly linked with oncogenesis.

Rather than doublet trials, Dr Jordi Rodón (Vall d’Hebron Instituto de Oncología, Spain) presented data from ODM-203, a novel kinase inhibitor that simultaneously targets tumour growth through fibroblast growth factor receptor (FGFR) and angiogenesis via vascular epithelial growth factor receptor (VEGFR). Compared to dedicated targeting of either receptor alone, preclinical data suggests that a two-pronged approach helps limit chemoresistance associated with monotherapy.

FGFR was also the target of research presented Dr Markus Joerger (Kantonsspital St. Gallen, Switzerland), in which a novel FGFR inhibitor BAY1163877 was administered in a dose escalation study to patients unusually stratified according to their mRNA expression. From this study, in which patients with advanced FGFR positive urothelial cancer had the best response, it could be taken that FGFR mRNA overexpression functions as a biomarker. Combined with recent advances in liquid biopsy and biomarker assaying, fluid samples from patients might be able to determine not only which type of cancer a patient has and where but also their suitability for this new class of drugs.

Similar multitargeted approaches were taken up by Prof Eric Van Cutsem (University of Leuven, Belgium) in the LUME trial utilising nintedanib against FGF VEGF, PDGF in colon cancer. While there was a PFS benefit, he reported no OS benefit resulting in a technically negative trial.

Similar results came from Prof Karim Fizazi (Institut Gustave Roussy, France) and Dr Alessandro Gronchi (National Cancer Institute, Milan, Italy). Prof Karim Fizazi and the AFFINITY trial found no significant survival improvements for castration resistant prostate cancer patients receiving custirsen. Dr Alessandro Gronchi, who assessed the impact of histology-based tailoring on adjuvant chemotherapy in soft tissue sarcoma patients, found an overall improvement for those receiving adjuvant chemotherapy compared to regular regimens but no improvement based on histology tailoring.

All three of these trials demonstrated a benefit for patients receiving them in some fashion but could not be counted as technical successes. Follow up studies are being planned for each to determine how to learn, refine, and improve upon their findings. For more on the value of negative trials, Dr Bishal Gyawali (Nagoya University, Japan) recently published an Editorial in *E*cancermedicalscience [6].

## Drug repurposing and replacement

A similar notion of salvaging clinical utility, not from failure but from the successes of other medications, was the focus of Dr Pan Pantziarka's (Anticancer Fund, UK) presentation with specific attention to propranolol.

Currently prescribed for high blood pressure and anxiety, among others, propranolol has already shown promise in paediatric haemangiomas in a wide selection of cell lines and animal models.

With a 100% response rate in angiosarcoma trials, propranolol seems to function against proliferation, angiogenesis, and immune modulation making it a prime agent in early cancer care if label extension is permitted.

However, the generic availability of the drug mean that no one producer is likely to take the low-return option of applying for said extension. This mirrors similar debates as when in the case of lobbying for aspirin repurposing.

Amidst the many advances being reported this year, Monday’s press conference had data that so neatly dovetailed, one presentation seemed to almost immediately supersede the other in line.

In the series of lectures, we first heard from Dr Alain Ravaud (Centre Hospitalier Universitaire de Bordeaux, France) about the results of phase III trial with adjuvant sunitinib for kidney cancer. The finding were that it improved DFS to 6.8 years with sunitinib compared to 5.6 years with placebo.

Immediately after, Dr Toni Choueiri (Dana Farber Cancer Institute, USA) introduced data from CABOSUN wherein it was observed a PFS benefit for patients with medium and poor-risk kidney cancer receiving cabozantinib over those receiving sunitinib. It was at 8.2 versus 5.6 months and an improvement in overall response (OR) (46% versus 18%) and in preliminary results of OS (30.3 versus 21.8 months).

Both authors were quick to caution against cross-trial comparisons, differences in patient selection, and that the sunitinib remains the standard of care for metastatic renal cell carcinoma.

It is reassuring, however, to know that the standard of care continues to extend its efficacy, and that the next line of treatment is gathering its own head of steam.

## FALCON has landed

Looking between the gaps of novel monotherapy and phase III trials, ESMO has also hosted results from innovative combinations of therapies. Some bridged the gap of mutationally specific targeted therapies with broad cytotoxics, and the others spoke on combining modalities. Among them, Dr Giorgio Scagliotti (University of Torino, Italy) gave results from the ASCEND-5 trial where ceritinib and docetaxel were used a rescue regimen in which criztonib had failed for ALK+ lung.

Ceritinib, a second generation ALK inhibitor, increased PFS and patient response almost five-fold, (5.4 versus 1.6 months, hazard ratio [HR] 0.49, p < 0.001, 39.1% versus 6.9%). While ALK mutations make up only a small number of lung tumours overall. These results are encouraging for those who have progressed after a first round of treatment, and it raises the question of bringing ceritinib to a standard of care in ALK+ cancer in the future.

This conference also featured results from the FALCON trial. Following the success of phase II FIRST [7] trials and matching the indications from the CONFIRM trial, a new standard of care could identified for HR+ breast cancer in fulvestrant. Compared to anastrazole, 500mg of fulvestrant, an endocrine agent, significantly increased PFS (16.6 months versus 13.8 months, p = 0.048). For those whose disease had not developed liver or lung metastases, the benefit was even greater (22.3 versys 13.8 months). These results echo the change of tamoxifen to aromatase inhibitors for breast cancer, and it open doors to a world of adjuvants and combination therapies, extending this survival advantage even further.

## CDK inhibitors: now and next

One such combination may follow up on the announcement made by Dr Gabriel Hortobagyi (MD Anderson, Houston, USA) who presented results of ribociclib alongside letrozole in the MONALEESA2 trial [8].

In the same patient subgroup as the FALCON trial (postmenopausal HR+ advanced breast cancer), patients in MONALEESA2 who received ribociclib, a CDK4/6 inhibitor, experienced a 44% improvement in PFS, meeting its primary end point early. Median PFS was 14.7 months in the placebo arm but was not reached in the ribociclib arm at data cut-off. Patients with measurable disease at baseline showed a significantly higher objective response rate to ribociclib plus letrozole compared to letrozole alone (53% versus 37%; p = 0.00028), and improved clinical benefit rate (80% versus 72% p = 0.02).

The results from this trial are indicative of a widening avenue of research and clinical development for cyclin-targeted treatment, which given the aforementioned successes of immune checkpoint therapy, could be considered as a sledgehammer of a checkpoint inhibitor – shutting down the cell cycle in its entirety.

Given that uncontrolled cell growth and division is the essence of cancer, a treatment that can halt these in their tracks would change the field of cancer care. Such hopes led to the hosting of a dedicated satellite symposium, hosted by ecancer and funded by Lilly, to summarise and debate the past, present, and future of therapeutic CDK4/6 inhibition. The session offered not only a learning opportunity for those in the audience, but also a direct method of feedback for presenters, with the audience members submitting their understanding of CDKs to the stage via live quiz responses. They used handheld devices to select from multiple choice questions to reinforce their own learning and give presenting doctors an impression of any areas that could need further discussion.

This system was trialled by the chair, Dr Giuseppe Curigliano (European Institute of Oncology, Milan, Italy), to set a base-level before Dr Ghadeer Shubassi (Institute of Molecular Oncology (IFOM), Milan, Italy) opened the session with a summary of the roles of CDK4 and 6 in cell biology and what makes them such opportune targets. He poised that the transitionary phase between G0 senescence and G1 in the cell cycle is a pause, a holding pattern of basic survival. Before signalling through Cyclin D kicks the cascade towards cell division into action, inhibition of CDK4/6 offers a chance to lock these cells into stasis. It halts their spread, slows tumour growth, and possibly even starves the high-metabolism cancer cells to an immunogenic death. That is the hope anyway. Clinical success is only now coming through with ribociclib in MONALEESA2 following on from PALOMA 1, 2, & 3 [9].

This history was summarised by Pierfranco Conte (Instituto Oncologico Veneto, Padua, Italy). He spoke on palbociclib explaining how it was tested alongside letrozole or fulverstrant for hormone positive advanced breast cancer, how palbociclib has shown to extend PFS for many, and finally how palbociclib now has taken firstline of therapy in US care. He stated that a clearly advantaged subgroup is yet to fully come out from these trials as there are other modalities taking precedence for some of these patients. He also said should those fail anyway, palbociclib has a place in combinations and second line therapy.

There may be many subgroup waiting in one of the many phase III trials as Dr Conte introduced, from PALLAS to PENELOPE to PALLET to the comparably plainly named LUM A + B studies. Another topic raised was that of side effects, an issue inherent to the somewhat leaky manipulation of cell cycle progression in tumours, with the most common being myelosuppression and neutropaenia.

Dr Conte reckoned these adverse events to be common to the whole class, considering similar outcomes from ribociclib in MONALEESA2, but also hastened to add that the effect is transient and comparably well tolerated. The ease in this tolerability may be because of the dosage in these trials with three weeks of treatment followed by one week of rest.

Even before formally following on from Dr Conte, Dr Sara Hurvitz (UCLA Medical Center, USA) considered whether this regimen may actually be allowing tumour cells time to recover, prolonging disease, and promoting eventual resistance. Weighing this against patient concerns and toxicity, it still may be some time before an answer is found there.

Taking to the podium for her session, Dr Hurvitz turned her focus to the statistically negative/clinically beneficial trial of abemaciclib, a potent CDK4 inhibitor, in the MONARCH1 trial in which patients receiving abemaciclib had a nearly 20% objective response rate, and asked why one thought neo-adjuvant was the place for CDK therapy. The short answer to this was because it works.

Beginning with slides identifying the most sensitive and resistant cell lines in clinical libraries, Dr Hurvitz introduced a valuable marker that may go some way to determining a predictive subgroup. The levels of Ki67, a protein associated with cell proliferation and disease aggressiveness, can be taken as a marker of response, and that the patients with a PI3KA mutation have demonstrated high levels of cell cycle arrest. While Ki67 does recover in the week off treatment, it can be re-suppressed with each subsequent treatment.

In practice, this looks like the outcomes of MONALEESA1, a precursor to the work presented by Dr Hortobagyi in which the admittedly small cohort of 14 patients were randomised to letrozole versus letrozole + ribociclib 400 versus letrozole + ribociclib 600 with those receiving ribociclib showing significant Ki67 suppression pre-surgery.

All this leads Dr Hurvitz to introduce NEOMONARCH, taking the success of abemaciclib into the neoadjuvant position, with even greater Ki67 modulation and less neutropaenia. Given the success of MONARCH [10] and the neoadjuvant schedule of MONALEESA1, NEOMONARCH will be yet another step forward for advanced breast cancer and open the door to biomarker discovery. Dr Hurvitz did note a slight increase in diarrhoea associated with abemaciclib over palbociclib, which she describes is easily managed by loperamide.

This altered toxicity profile is the launching point from which the last symposium speaker, Dr Sybil Loibl (German Breast Cancer Group), began. She noted a lower grade neutropaenia in comparison and also noted other variations in adverse events that set abemaciclib apart from ribociclib and palbociclibs, the near-identical profile. Looking at CDK therapy in a broader view, she went onto consider the alternative courses of treatment for patients, with the most significant other modality being endocrine therapy.

Framing endocrine therapy through the recently announced FALCON study, which treated a comparable though not wholly identical population, Dr Loibl pitched the PALOMA3 study of palbociclib plus fulvestrant as combined treatment extending PFS by four to nine for high risk patients. BOLERO4, on the other hand, tested letrozole plus everolimus resulting in patient benefit in the single-arm study, and Dr Loibl mused that adding a CDK to this mix might offer even greater impact still.

She also considered the prognostic value of ESR1, a mutational target, identified through the SoFEA trial that indicated susceptibility to fulvestrant depending on prior treatment but was not especially indicative for palbociclib response.

Noting that there is still a place for chemotherapy, Dr Loibl summarised with a side by side comparison of PFS from endocrine monotherapy versus combination with a CDK, with additional palbociclib or ribociclib both edging letrozole further towards a year’s worth of PFS. Out ahead by far is fulvestrant, leading Dr Loibl to the claim that for treatment naive patients, fulvestrant plus CDK4/6 is the optimal therapy. She also expects that monoendocrine therapy usage will diminish, and should a suitable biomarker be uncovered, CDK4/6 inhibitors will have a key role to play in future breast cancer therapy.

## Conclusion

With those last thoughts of the last session on the last day, another conference drew to a close. We have seen checkpoint inhibition move from promise to delivery, previously cold tumour sites growing warmer, and exciting new data for fledgling therapies in PARP and CDK inhibitors in this conference. There were only a handful of speakers in the conference, but there are dozens of other trials being reported upon and few other data yet to mature. For those, we will have to keep an ear to the ground in future conferences and see what awaits us in 2017’s conference cycle.

## References

[1] Reck M (2016). Pembrolizumab versus chemotherapy for PD-L1–positive non–small-cell lung cancer. N Engl J Med.

[2] Langer Corey J (2016). Carboplatin and pemetrexed with or without pembrolizumab for advanced, non-squamous non-small-cell lung cancer: a randomised, phase 2 cohort of the open-label KEYNOTE-021 study. Lancet Oncol.

[3] Eggermont AM (2016). Prolonged survival in stage III melanoma with ipilimumab adjuvant therapy. New Engl J Med.

[4] Mirza MR (2016). Niraparib maintenance therapy in platinum-sensitive, recurrent ovarian cancer. N Engl J Med.

[5] Bang YJ (2015). Randomized, double-blind phase II trial with prospective classification by ATM protein level to evaluate the efficacy and tolerability of olaparib plus paclitaxel in patients with recurrent or metastatic gastric cancer J Clin Oncol.

[6] Gyawali B, Prasad V (2016). Negative trials in ovarian cancer: Is there such a thing as too much optimism?. Ecancermedicalscience.

[7] Ellis MJ (2015). Fulvestrant 500 mg versus anastrozole 1 mg for the fir*st*-line treatment of advanced breast + phase II FIRST study. J Clin Oncol.

[8] Hortobagyi CN (2016). Ribociclib as first-line therapy for HR-Positive, advanced breast cancer. N Engl J Med.

[9] Lim JS (2016). CDK4/6 inhibitors: promising opportunities beyond breast cancer. Cancer Discov.

[10] https://clinicaltrials.gov/ct2/show/NCT02102490?term=MONARCH+1&rank=1.

